# Reducing Th2 inflammation through neutralizing IL-4 antibody rescues myelination in IUGR rat brain

**DOI:** 10.1186/s11689-019-9297-6

**Published:** 2019-12-16

**Authors:** Allison E. Zanno, Micah A. Romer, Lauren Fox, Thea Golden, Lane Jaeckle-Santos, Rebecca A. Simmons, Judith B. Grinspan

**Affiliations:** 1 Department of Pediatrics, Children’s Hospital of Philadelphia, Perelman School of Medicine at the University of Pennsylvania, Philadelphia, PA USA; 2 Department of Neurology, Children’s Hospital of Philadelphia, Perelman School of Medicine at the University of Pennsylvania, 516D Abramson Center, 3615 Civic Center Blvd, Philadelphia, PA 19104 USA

**Keywords:** IL-4, Myelin, Oligodendrocyte, Inflammation

## Abstract

**Background:**

Intrauterine growth restriction (IUGR) is a common complication of pregnancy and is associated with significant neurological deficits in infants, including white matter damage. Previous work using an animal model of IUGR has demonstrated that IUGR rats exhibit neurobehavioral deficits and developmental delays in oligodendrocyte maturation and myelination, but the mechanisms which cause this delay are unknown. Inflammation may be an important etiological factor in IUGR and has been recognized as playing a fundamental role in the pathogenesis of myelin disorders, including cerebral palsy.

**Methods:**

To create the model, the uterine arteries of pregnant rats were ligated at embryonic day 15. Rats delivered spontaneously. Cytokine and chemokine expression was evaluated at one prenatal and three postnatal time points, and myelin protein expression and oligodendrocyte cell numbers were evaluated by several methods at postnatal day 14. IL-4 was identified as a potential inhibitor of myelination, and rat pups were injected with IL-4 function blocking antibody from postnatal days 1–5 and myelination was assessed.

**Results:**

Here, we show a novel mechanism of white matter injury. IUGR induces an exaggerated Th2 response in the developing rat brain, including upregulation of several Th2 cytokines. Of these, IL-4 is significantly increased during the period corresponding to robust developmental myelination. We show that neutralizing IL-4 antibody therapy given in the newborn period ameliorates inflammation and restores myelin protein expression and oligodendrocyte cell number in the IUGR brain to control levels, demonstrating a novel role for Th2 responses and IL-4 in IUGR and white matter injury. In addition, IL-4 directly affects oligodendrocytes in vitro decreasing differentiation.

**Conclusions:**

In this study, we have identified inflammation as a factor in the decrease in myelin seen in an animal model of IUGR. IL-4, an inflammatory protein often thought to be protective in the adult, is specifically increased, and treatment of these animals to prevent this increase ameliorates white matter damage. Our results suggest that the immune system plays a role in IUGR that is different in the perinatal period than in the adult and preventing this exaggerated Th2 response may be a potential therapeutic target.

## Background

Intrauterine growth-restricted (IUGR) newborns face high rates of neonatal mortality and morbidity [1] including neurological deficits ranging from behavioral and motor disabilities to cerebral palsy [2–4]. White matter injury is common in these infants and is characterized by a lack of mature oligodendrocytes and myelin. Oligodendrocyte progenitors (OPCs) are unable to differentiate and are arrested in an immature state, resulting in a lack of myelin and the susceptibility to further damage [5, 6]. Identifying the factors that block this differentiation would allow us to devise therapies to direct oligodendrocytes to complete their maturation process, protecting them from further damage. Currently, there are no such therapies available to mitigate the white matter injury in these babies.

One potential target for future therapies is the inflammatory process mediated by cytokines. Inflammation has already been recognized as playing a fundamental role in the pathogenesis of myelin disorders. Inflammation in the brain can be mediated through microglia/macrophages, the resident macrophages of the CNS. Inflammatory cells such as macrophages and T cells may also invade the newborn brain to cause damage. Clinical evidence has shown that growth-restricted newborns are at an increased risk for systemic inflammation. Growth-restricted newborns at 2 weeks of age are significantly more likely than their counterparts to have elevations in inflammatory proteins such as CRP, IL-6, MCP, and TNF-alpha that cannot be attributed to delivery mode, severity of illness, bacteremia, or duration of ventilation [7]. Cytokine levels are also altered in cord blood of preterm infants who are growth restricted as compared to appropriate for gestational age newborns [7, 8].

In addition to white matter injury, multiple studies have shown growth-restricted newborns are at increased risk for the development of obesity, type 2 diabetes, asthma, allergies, and the metabolic syndrome later in life [9, 10]. Inflammation may be one unifying mechanism underlying the increase in incidence of these diseases in individuals who were born IUGR. In previous studies, we found that Th2 cytokines are significantly increased in islets in an animal model of IUGR, which leads to injury of β cells and endothelial cells [11]. A Th2 response in the newborn is not unexpected since immune responses in the normal fetus and newborn are usually skewed towards a Th2 phenotype allowing for maternal immune tolerance [12–14]. However, in the setting of IUGR, this response is markedly exaggerated and leads to injury rather than preventing injury.

IL-4 is a key Th2 cytokine and has been extensively studied in adult models of stroke and multiple sclerosis where it is thought to be neuroprotective and anti-inflammatory [15]. However, very little data exist on the role of IL-4 in the fetal and neonatal brain, although it is known that IL-4 is expressed in higher amounts in the neonatal brain than in the adult brain and is thought to be produced endogenously [16]. Under normal conditions, the production of IL-4 in the periphery is limited to a few cell types including mast cells, macrophages, T cells, eosinophils, and basophils [17]. In the brain, resident microglia/macrophages are the most likely candidates [18] although astrocytes are also capable of producing IL-4 [19]. Furthermore, a number of cells in the brain have IL-4 receptors including oligodendrocytes and microglia/macrophages [8].

In this study, we used our well-characterized rat model of intrauterine growth restriction (IUGR) which we have previously shown to cause delayed oligodendrocyte maturation and myelination [5]. Here, we demonstrate that IUGR induces a Th2 inflammatory response in rat brain. This Th2 response leads to an increase in IL-4 and resultant damage to OPCs leading to white matter injury. This injury can be rescued/prevented by IL-4 neutralizing antibody demonstrating that inflammation in an IUGR model, mediated by IL-4, leads to white matter loss.

## Methods

### Animal model

All experiments were performed in accordance with the guidelines set forth by The Children’s Hospital of Philadelphia Institutional Animal Care and Use Committee. Our animal model has been previously described [5, 20]. Briefly, Sprague-Dawley pregnant rats (Charles River Laboratories, Wilmington, MA) were individually housed under standard conditions and allowed free access to standard rat chow and water. On day 17 of gestation (term is 22 days), the maternal rats were anesthetized with inhaled isofluorane and both uterine arteries were ligated. Rats were allowed to recover and had ad lib access to food and water. The pregnant rats were allowed to deliver spontaneously, and the litter size was reduced to 8 at birth to assure uniformity of litter size between IUGR and control litters. Measurements were made using both male and female pups at embryonic day 19 (e19), postnatal day 1(PD1), postnatal day 7 (PD7), and postnatal day 14 (PD14).

### Cytokine measurements

Unperfused brain tissue was collected and suspended in lysis buffer (PBS with 0.1% Triton-X 100 (Integra), 1% Protease Inhibitor Cocktail (Sigma), and 1% Phosphatase Inhibitor Cocktail (Sigma)). Tissues were homogenized, sonicated, and centrifuged for 10 min at 10,000 rpm. Lysate samples were normalized to total protein concentration as measured by BCA assay (Pierce). Cytokine and chemokine panels were measured by Luminex assay (EMD Millipore). The following 25 cytokines and chemokines were analyzed: IL-2, IL-4, IL-5, IL-6, IL-10, IL-13, IL-18, eotaxin, MCP-1, GRO-KC, leptin, IL-1a, IL-1b, 1 L-17a, IL-12p70, GM-CSF, MIP-1a, IFN-g, VEGF, fractaline, MIP-2, TNF-alpha, rantes, IP 10, and G-CSF.

### Perfusion and histology

To prepare sections of IUGR and sham brains, rats were killed at postnatal day (PD) 14 by perfusion in 4% paraformaldehyde. Frozen sections were prepared and cut on a Leica cryostat at 12-μm thicknesses, all according to established protocols [21, 22]. To label mature oligodendrocytes, we used anti-myelin proteolipid protein (PLP) (1:2, rat hybridoma [23]) and anti-CC1, which labels oligodendrocyte cell bodies (CC1, 1:20, Millipore, Billerica, MA). For microglia/macrophages, we used anti-IBA-1 (Wako Pharmaceuticals, 1:1000) and anti-CD68 (Abcam, 1:100). For IL-4 receptor, we used an anti-IL-4R (Abcam, 1:50). Secondary antibodies of appropriate species and isotype used for external and internal antigens were purchased from Jackson Immunoresearch, West Grove, PA. Coverslips were mounted over the sections in 4′,6-diamidino-2-phenylindole (DAPI)-containing Vectashield mounting medium (Vector Laboratories, Burlingame, CA, USA).

To count cells from frozen sections, IUGR and sham animals from at least three litters were used. Digital images were taken at × 20 magnification from sections at the level of the anterior part of the corpus callosum, counting 20,150 μm^2^ regions of interest per section, at least two sections per animal. Statistical significance was calculated using Student’s *t* test.

### Western blotting

Cell extracts were prepared from PD14 whole rat brain (excluding the hind brain) in ice-cold tissue extraction buffer as previously described [5], followed by centrifugation at 14,000 rpm at 4 °C for 30 min. Protein concentrations of collected supernatants were determined by a NanoDrop spectrophotometer. Ten to 25 μg of protein was loaded into each lane of 4–12% Bis-Tris gradient gel for separation. For detection of PLP, gels were run under non-reducing conditions due to antibody specificity. A broad spectrum molecular weight ladder was run on each gel. Following separation, proteins were transferred onto Millipore Immobilon-FL membranes and blocked in TBS with 0.1% Tween-20 (PBST) and 5% milk for 30 min at 4 °C. Membranes were incubated overnight at 4 °C with primary antibodies in TBST + 5% BSA. Membranes were incubated with the following primary antibodies: anti-myelin basic protein (MBP, rat hybridoma supernatant, 1:1000), anti-proteolipid protein (PLP, rat hybridoma supernatant, 1:1000), anti-CNP (Abcam, 1:1000), and anti-GFAP (rat hybridoma, 1:5000). All secondary antibodies were conjugated with IrDye at either 680 or 800 (LI-COR, Odyssey) and used at 1:10,000. Membranes were washed with PBST, and incubated with corresponding antigen-specific fluorescent probe-conjugated secondary antibodies (1,10,000 dilution) in TBST + 5% BSA. The membranes were imaged using Odyssey (Li-Cor). Blots were additionally probed for glyceraldehyde 3-phosphate dehydrogenase (GAPDH; 1:8000, Chemicon International) or tubulin (1:10,000, Sigma, St. Louis, MO) as a loading control for protein quantification. Bands of interest were specified to determine pixel intensities for each treatment using Licor Odyssey Software (Lincoln Nebraska), and the band intensities were normalized to loading controls to ensure equal loading. Statistical significance for the protein quantification was calculated using Student’s *t* test.

### Neutralizing IL-4 therapy

Control and IUGR animals were injected subcutaneously with 0.05 μg of purified mouse anti-rat IL-4 antibody (BD Pharmingen) or PBS (Fisher BioReagents) daily from postnatal days 1–5 as previously described [11]. At postnatal day 14, either rats were perfused for immunohistochemistry or brains were collected and frozen for immunoblotting.

### Cell culture generation and treatment

To generate cultures of purified OPCs from newborn rats, a mixed population of cells was harvested from the neonatal brain and seeded on 75-mL polylysine-coated flasks containing Neurobasal medium (Invitrogen, Life Technologies, Grand Island, NY) as previously described [5]. After 24 h, the cell cultures were switched to a serum-free growth medium containing Neurobasal medium (Invitrogen, Life Technologies, Grand Island, NY) with B27 supplement (1:50; Life Technologies), 10 ng/ml basic fibroblast growth factor, 2 ng/ml platelet-derived growth factor (both from R&D Systems), and 1 ng/ml neurotrophin-3 (Peprotech, Rocky Hill, NJ). Cultures were purified using modifications of a shake-off procedure, and purity varied between 90 and 99% OPCs [24]. Once confluent, the cells were subcultured into 12-mm polylysine-coated coverslips for immunofluorescence or 100-mm polylysine-coated Petri dishes for Western blotting.

To determine the ability of OPCs to differentiate, cultures were established at P1 as described above and grown until 80% confluent, approximately 1 week. Some cultures were collected at this point for non-differentiated controls. Growth medium was removed from cultures, and cells were fed with “differentiation medium” (DM), consisting of 50% Dulbecco’s modified eagle medium, 50% Ham’s F12 with 50 μg/ml transferrin, 5 μg/ml putrescine, 3 ng/ml progesterone, 2.6 ng/ml selenium, 12.5 μg/ml insulin, 0.4 μg/ml T4, 0.3% glucose, 2 mM glutamine, and 10 ng/ml biotin. Plates and coverslips were treated with either no IL-4 (Abcam) or varying concentrations of IL-4 and collected at 72 h after treatment.

### Immunocytochemistry

Cells on coverslips were processed for detection of specific antigens as described previously [22]. Oligodendrocytes were detected using anti-galactocerebroside antibody (GalC, RmAb, 1:1, [25]). Secondary antibodies of appropriate species and isotype were purchased from Jackson Immunoresearch, West Grove, PA. Coverslips were mounted onto glass slides in 4′,6-diamidino-2- phenylindole (DAPI)-containing Vectashield mounting medium (Vector Laboratories, Burlingame, CA, USA).

To count cells expressing antigens in culture, antigen-positive and DAPI-positive cells were counted in 20 fields in each of three coverslips from at least three separate preparations of cells using a Leica DM6000B fluorescence microscope at × 40 magnification. Statistical significance was calculated using Student’s *t* test.

## Results

### IUGR brain shows an increase in localized Th2 response

**Fig. 1:**
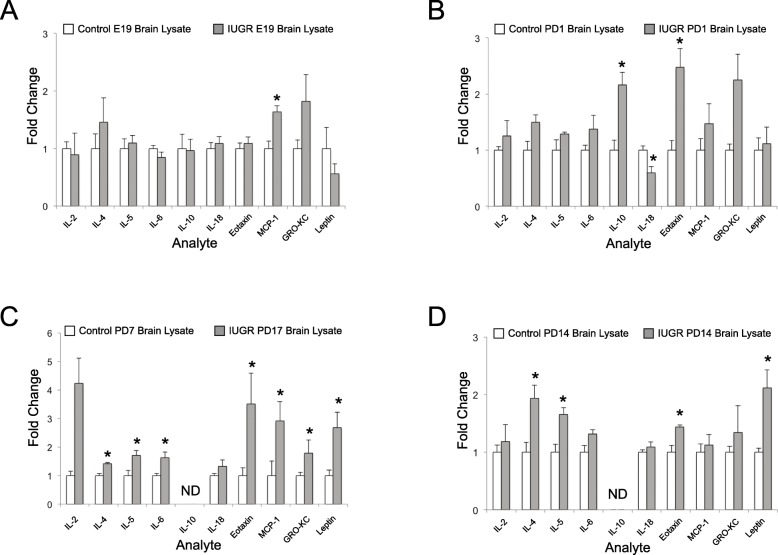
Specific cytokines and chemokines are significantly elevated in IUGR brains during development. Inflammatory cytokines and chemokines were measured by Luminex assay in total brain lysate at **a** e19, **b** PD1, **c** PD7, and **d** PD14. Data are expressed as fold change compared with controls (*n* = 4 measurements/age/group). Those with a statistically significant difference at a minimum of one time point are shown. *Significant difference versus control [**a** MCP 1: *P* < 0.001; **b** IL-10: *P* = 0.004, IL-18: *P* = 0.021, eotaxin: *P* = 0.007; **c** IL-4: *P* = 0.028, IL-5: *P* = 0.019, IL-6: *P* = 0.020, eotaxin: *P* = 0.038, Gro-KC: *P* = 0.028, leptin: *P* = 0.032; **d** IL-4: *P* = 0.011 m IL-5: *P* = 0.010, eotaxin: *P* = 0.010, leptin: *P* = 0.012, two-tailed *T* test]; ND, undetectable; white bars, control; gray bars, IUGR

To characterize immune activation in the IUGR brain, we measured 25 inflammatory cytokines and chemokines in the isolated rat brain using a Millipore Luminex Panel. This panel contains both T1 and T2 immune cytokines and chemokines. Cytokine profiling of the isolated IUGR and control rat brain was assessed at embryonic day 19 (e19), postnatal day 1 (PD1), postnatal day 7 (PD7), and postnatal day 14 (PD14) (Fig. 1). Cytokines and chemokines with statistical significance at any time point are shown. At e19 (Fig. 1a), only the chemokine MCP-1 (monocyte chemotactic protein) was elevated which recruits monocytes, memory T cells, and dendritic cells to sites of injury. On PD1 (Fig. 1b), inflammation started to increase and more chemokines and cytokines were elevated including eotaxin and IL-10. At PD7 (Fig. 1c), inflammation peaked with significant increases in eotaxin, IL-2, IL-4, Il-5, leptin, IL-4, MCP-1, and Gro-KC. Finally, at PD14 (Fig. 1d), inflammation persisted but started to decrease and only eotaxin, IL-2, Il-4, IL-5, and leptin levels were elevated. This demonstrated multiple different inflammatory markers were elevated at various time points in IUGR rats beginning at PD1, peaking at PD7, and subsiding at PD14. This time frame corresponded to the oligodendrocyte pathology previously seen with peaks between PD7 and PD14 [5].

### IUGR brain has increased inflammation as shown by an increase in microglia/macrophages

**Fig. 2:**
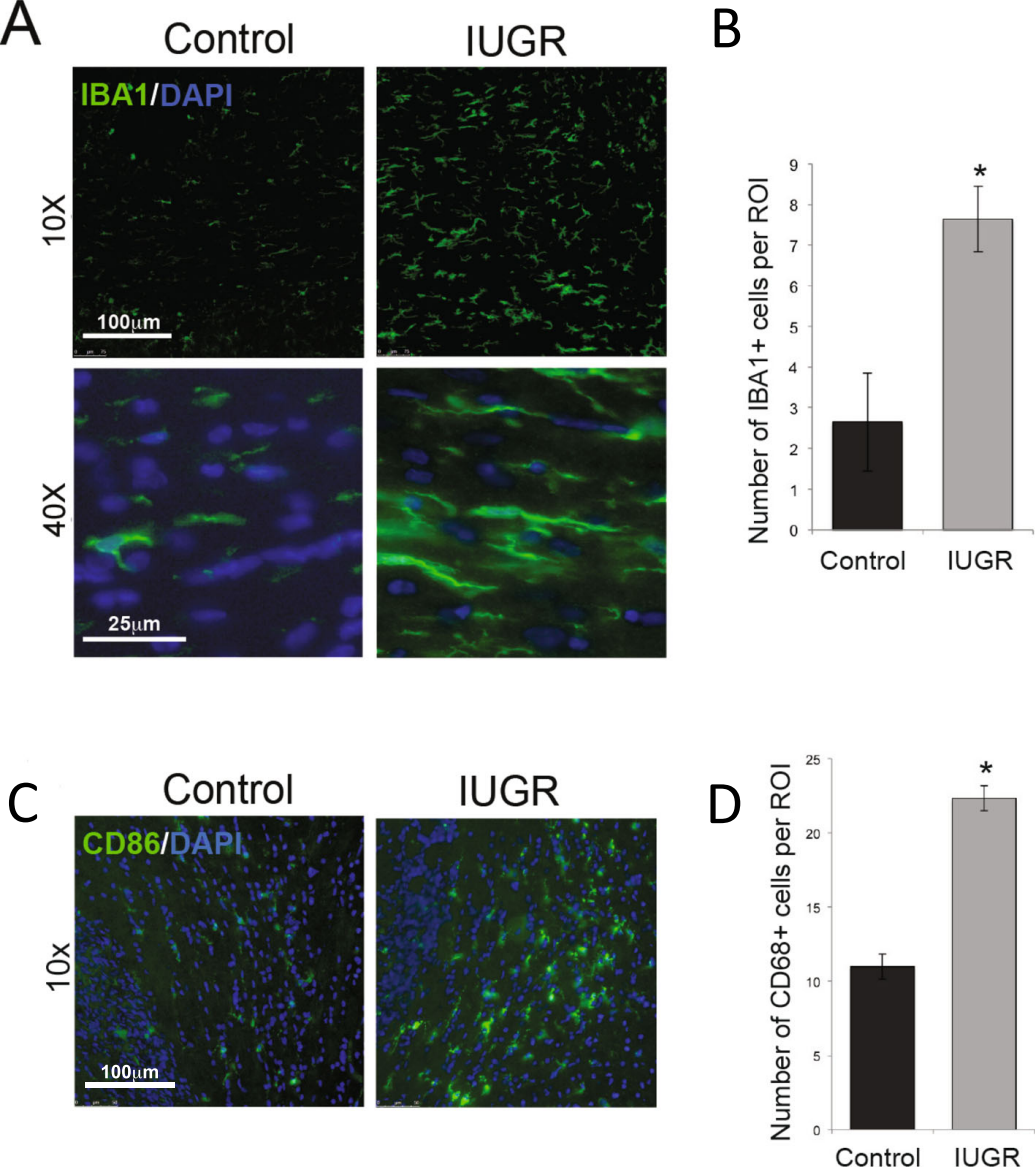
Microglia/macrophages are increased in the IUGR brain at P14. Sections of the IUGR and control brain at P14 were labeled with antibody to the microglia/macrophages marker IBA1 or activated microglia/macrophages marker CD68 as well as DAPI to identify nuclei. **a** Shown are sections of the corpus callosum from control and IUGR rat brains labeled with IBA1 (green) and DAPI (blue) at PD14 at × 10 and × 40 magnification (size bars as indicated). Microglia/macrophages are more numerous and appear larger. **b** Quantification of IBA1+ microglia/macrophages per ROI (*n* = 4–5 pups per condition). *Significant difference *P* = 0.011 versus control, two-tailed *T* test. **c** Shown are sections of control and IUGR rat brains at P14 labeled with CD68 (green) and DAPI (blue) and photographed at × 10 at the genu of the corpus callosum. Activated microglia/macrophages were significantly increased in this location. **d** Quantification of CD68+ microglia/macrophages per ROI (*n* = 3 pups per condition). *Significant difference *P* ≤ 0.001 versus control, two-tailed *T* test

In order to examine the brain for evidence of inflammation, we labeled sections of the PD14 corpus callosum with IBA1 antibody which labels microglia/macrophages (Fig. 2). In IUGR animals, the microglia/macrophages in the corpus callosum appeared larger and more abundant when compared with sham animals (Fig. 2a). When this difference was quantified, there was almost a 2.5-fold increase in microglia/macrophages in IUGR animals in the corpus callosum compared with control animals (Fig. 2b). We also looked for evidence of activated microglia/macrophages by CD68 staining (Fig. 2c). This was most evident in the genu of the corpus callosum where there was a greater than twofold increase in activated microglia/macrophages (Fig. 2d) compared with control.

### IL-4 neutralizing antibody decreases IL-4 in vivo

**Fig. 3:**
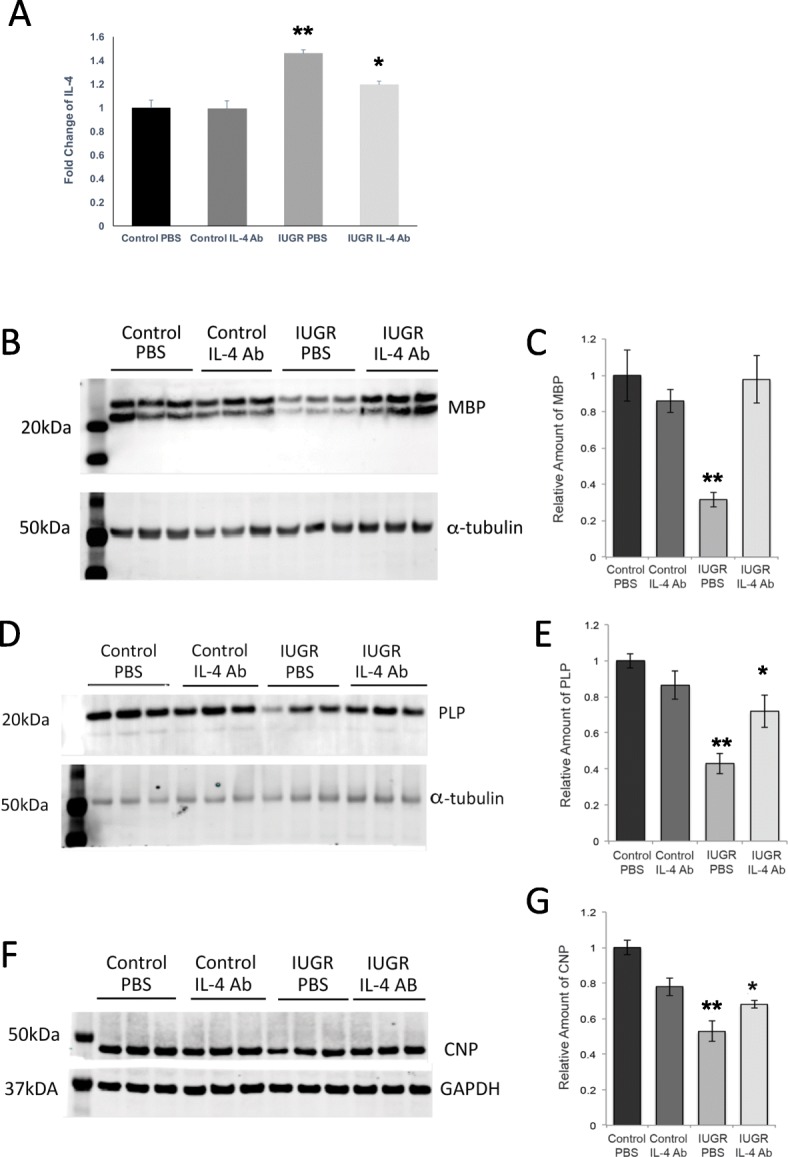
Injection of IL-4 blocking antibody reverses increases in IL-4 protein and decreases in myelin proteins in IUGR. **a** IUGR and control pups were injected with IL-4 neutralizing antibody or PBS from days 1–5, and total brain lysates were collected at P14. IL-4 levels were measured by Luminex assay. Data are expressed as fold change compared with controls (*n* = 5 measurements/age/group). *Significant difference at *P* < 0.05 versus control PBS, two-tailed *T* test. **Significant difference at *P* < 0.05 versus IUGR PBS, two tailed *T* test. **b** Expression of myelin basic protein (MBP) was assessed at P14 in IUGR and control pups treated with anti-IL-4 or PBS from days 1–5. MBP and loading control (beta-tubulin) expressions are shown from three animals for each treatment type and demonstrate significantly lower of MBP expression in PBS-treated IUGR pups increasing to normal levels after anti-IL-4 treatment. **c** Western blot quantification relative to loading control also shows normalization of MBP expression in brain lysates from anti-IL-4-treated IUGR pups at P14. *Significant difference at *P* = 0.0009 versus control PBS, two-tailed *T* test. **Significant difference at *P* = 0.0006 versus IUGR PBS, two-tailed *T* test. **d** and **e** Proteolipid protein (PLP) *Significant difference at *P* = 0.00002 versus control PBS, two-tailed *T* test. **Significant difference at *P* = 0.031 versus IUGR PBS, two-tailed *T* test. **f** and **g** 2′,3′-Cyclic-nucleotide 3′-phosphodiesterase (CNP) expression levels are likewise significantly lower in PBS-treated IUGR pups, increasing to near normal levels in anti-IL-4-treated animals. *N* = 6 pups per group for all myelin proteins. *Significant difference at *P* = 0.00012 versus control. **Significant difference at *P* = 0.041 versus IUGR PBS, two-tailed *T* test

The IL-4 cytokine levels were elevated on the brain Luminex screens at the time point corresponding to the height of myelination. Although the brains were not perfused, plasma from IUGR pups has been tested separately and IL-4 was not elevated at e19 or PD14 [11]. Although IL-4 is thought to be protective, anti-IL-4 injection into newborn IUGR pups was shown to rescue a pancreas/diabetic phenotype in the adult IUGR rat, and control IgGs used in those experiments had no effect [11]. Given that oligodendrocytes have IL-4 receptors [15], we hypothesized that reduction of IL-4 may improve myelination. We selected an IL-4 neutralizing antibody and first performed a Luminex assay in order to determine if the IL-4 neutralizing antibody decreased IL-4 levels in vivo. Neutralizing IL-4 antibody decreased IL-4 levels to control levels in vivo at PD14 (Fig. 3a), indicating the effectiveness of the IL-4 neutralizing antibody. We also examined signaling in the Luminex screen to see if IL-4 neutralizing antibody affected other cytokines. Only leptin was significantly altered by IL-4 neutralizing antibody treatment. The other 23 cytokines were not affected (data not shown).

### IL-4 neutralizing antibody decreases microglia/macrophages in vivo

**Fig. 4:**
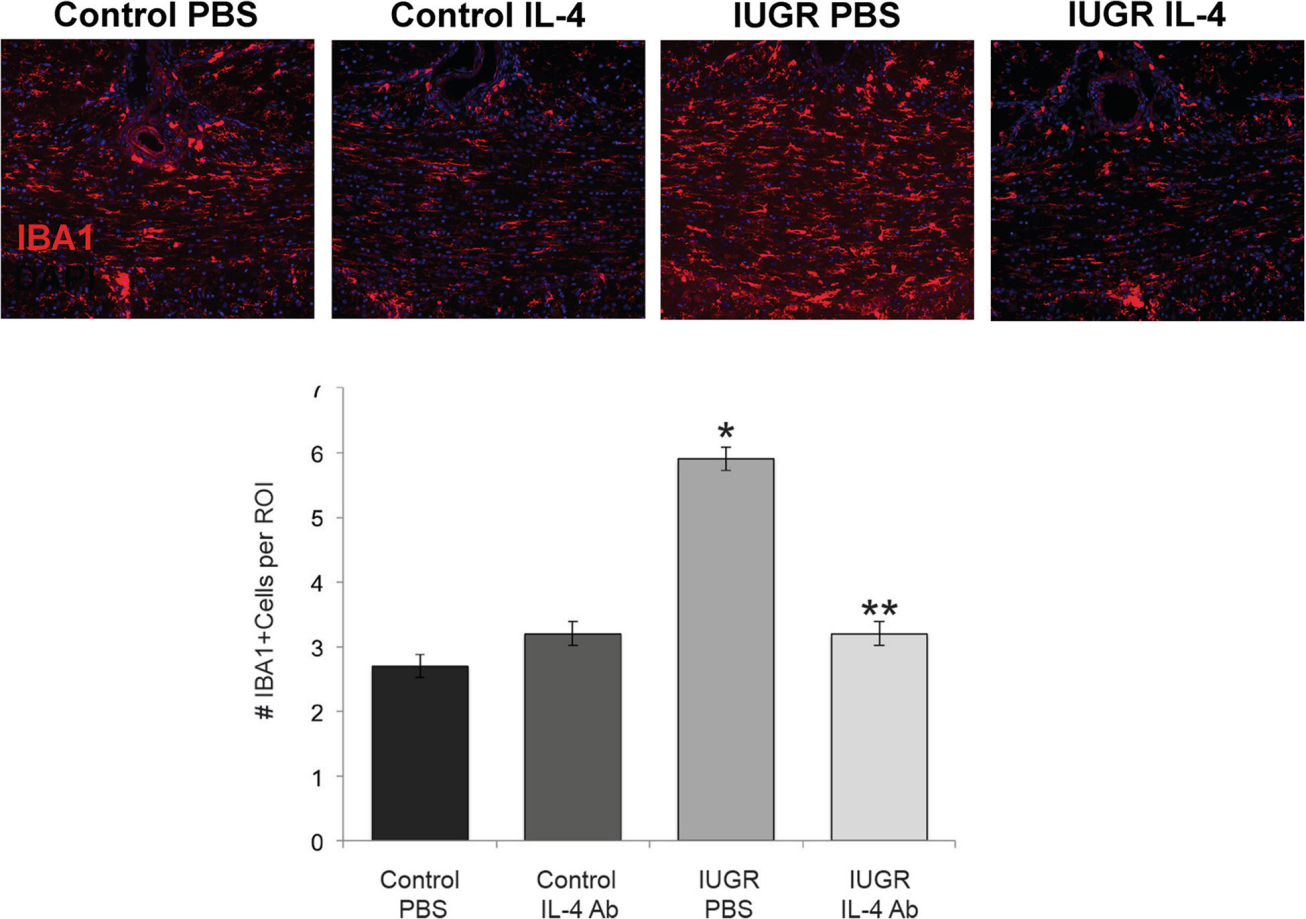
Microglia/macrophages return to control levels with injection of IL-4 neutralizing antibody. Sections of IUGR and control rat brains injected with IL-4 neutralizing antibody or PBS from days 1–5 were labeled with IBA1 antibody to identify microglia/macrophages. Quantification of IBA+ cells per ROI in corpus collosum (*n* = 3 rats per condition) demonstrates that microglia/macrophage levels decrease after anti-IL-4 injection. *Significant difference at *P* = 0.002 versus control PBS, two-tailed *T* test*.* **Significant difference at *P* < 0.015 versus IUGR PBS, two-tailed *T* test

Since IL-4 was restored to control levels, we wanted to determine if the number of microglia/macrophages was also restored to normal. We therefore labeled sections of the frozen rat brain treated with either PBS or IL-4 neutralizing antibody and counted the number of IBA1+ cells in the corpus collosum. IL-4 neutralizing antibody, compared with PBS, decreased the number of microglia/macrophages in IUGR animals to control level (Fig. 4).

### IL-4 neutralizing antibody restores myelination in vivo

In order to test whether IL-4 neutralizing antibody abrogates the white matter injury seen in IUGR, we injected control and IUGR pups subcutaneously on PD1–5 with either IL-4 neutralizing antibody or PBS. At PD14, we performed Western blots for myelin proteins myelin basic protein (MBP), proteolipid protein (PLP), and 3′,4′-cyclic nucleotide phosphodiesterase (CNP) (Fig. 3b–d). In the PBS-treated IUGR pups, we saw the characteristic decrease in MBP to as little as 30% of control levels (Fig. 3b, c [5]). We have shown that this is due to reduced maturation as numbers of OPCs were equal between IUGR and control in our previous studies [5]. Here, MBP levels in pups injected with IL-4 neutralizing antibody were restored to normal (Fig. 3b, c). PLP and CNP were also significantly decreased but not as severely (40% and 55%, respectively). The IL-4 neutralizing antibody restored myelination to 72% and 68% of control levels, respectively (Fig. 3d-g).

**Fig. 5:**
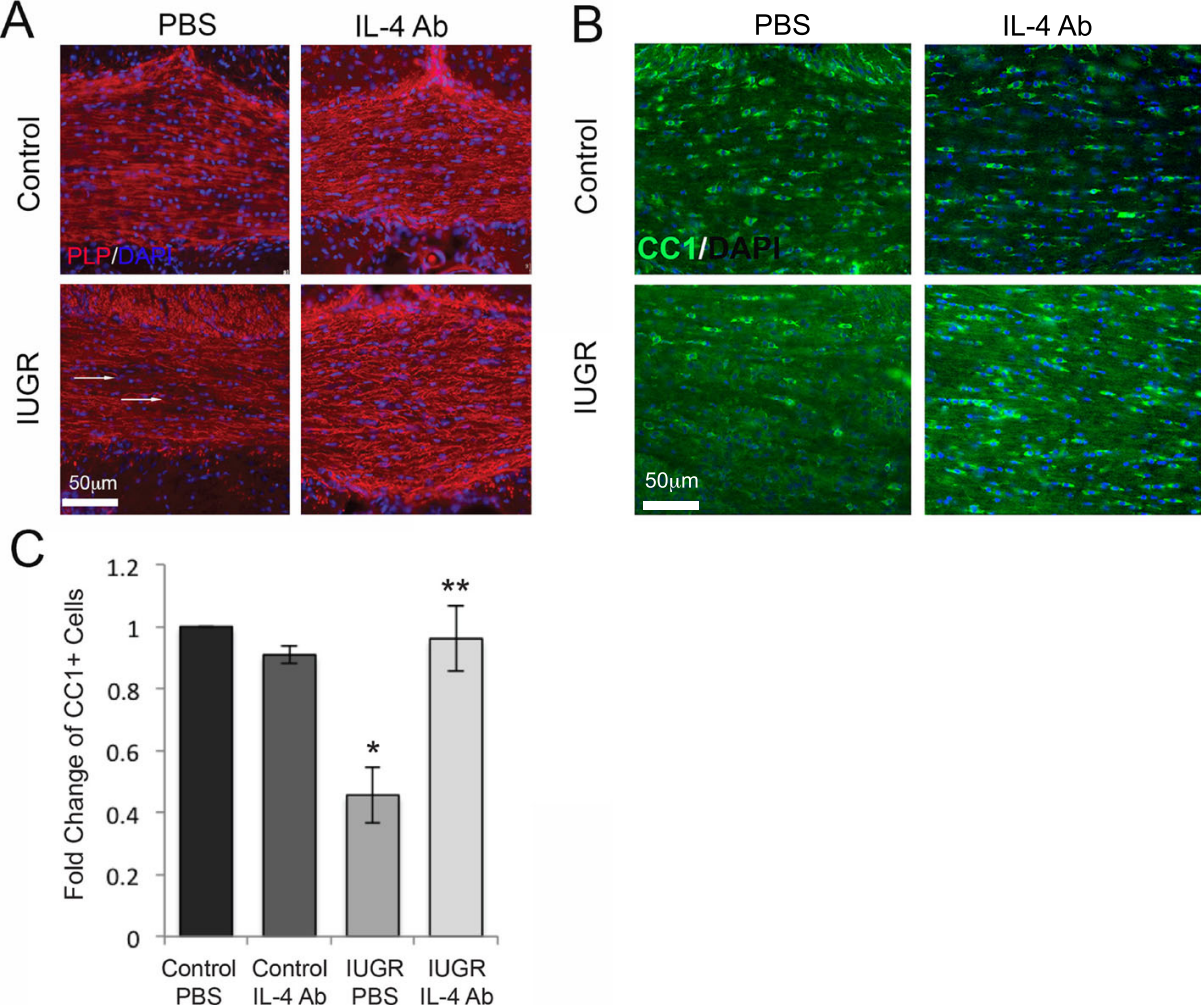
Injection of IL-4 blocking antibody increases numbers of oligodendrocytes in IUGR to control levels. **a** Shown are representative sections of the P14 corpus callosum from control and IUGR pups treated with anti-IL-4 or PBS labeled with antibody to PLP. DAPI, blue; PLP, red. Arrows indicate areas of patchy myelin loss in IUGR animals treated with PBS which are not evident in anti-IL-4-treated IUGRs. **b** Shown are representative sections of the P14 corpus callosum from control and IUGR pups treated with anti-IL-4 or PBS stained with antibody to with CC-1, which labels the cell body of mature oligodendrocytes. DAPI, blue; CC1, green. **c** Quantification of CC1+ cells per ROI in the corpus callosum demonstrates that the PBS-treated IUGRs have significantly fewer oligodendrocytes than control or anti-IL-4-treated IUGR section (*n* = 3 rats per condition). *Significant difference at *P* = 0.017 versus control PBS, two-tailed *T* test. **Significant difference at *P* = 0.05 versus IUGR PBS, two-tailed *T* test

In order to visualize the effect of anti-IL-4 oligodendrocytes and myelin in the corpus callosum in vivo, we labeled sections with antibody to PLP (Fig. 5). PLP staining showed the patchy lack of myelin previously identified in IUGR animals [5], which appeared to be rescued by treatment with IL-4 neutralizing antibody (Fig. 5a).

It is possible that IUGR leads to less myelination and IL-4 neutralizing antibody restores quantity of myelin without affecting the total number of oligodendrocytes. We therefore labeled sections of the PD14 corpus callosum with CC-1 antibody, which stains oligodendrocyte cell bodies and DAPI to label nuclei (Fig. 5b). The numbers of CC1+ and DAPI+ nuclei were counted per region of interest, and a percentage of CC1+ oligodendrocytes were normalized to the number of DAPI+ cells per region of interest (ROI). There was no significant difference in the number of DAPI+ cells per RO1 in controls versus IUGR, indicating that the IUGR corpus callosum had the same cell density as the controls. However, IUGR animals have approximately 50% of the oligodendrocytes in the corpus callosum in the IUGR animals (Fig. 5c), and this number increased to normal levels in animals treated with IL-4 neutralizing antibody.

Of importance, IL-4 neutralizing antibody had no effect on myelin proteins, myelination, and oligodendrocyte numbers in control animals.

### IL-4 inhibits oligodendrocyte differentiation in vitro

**Fig. 6:**
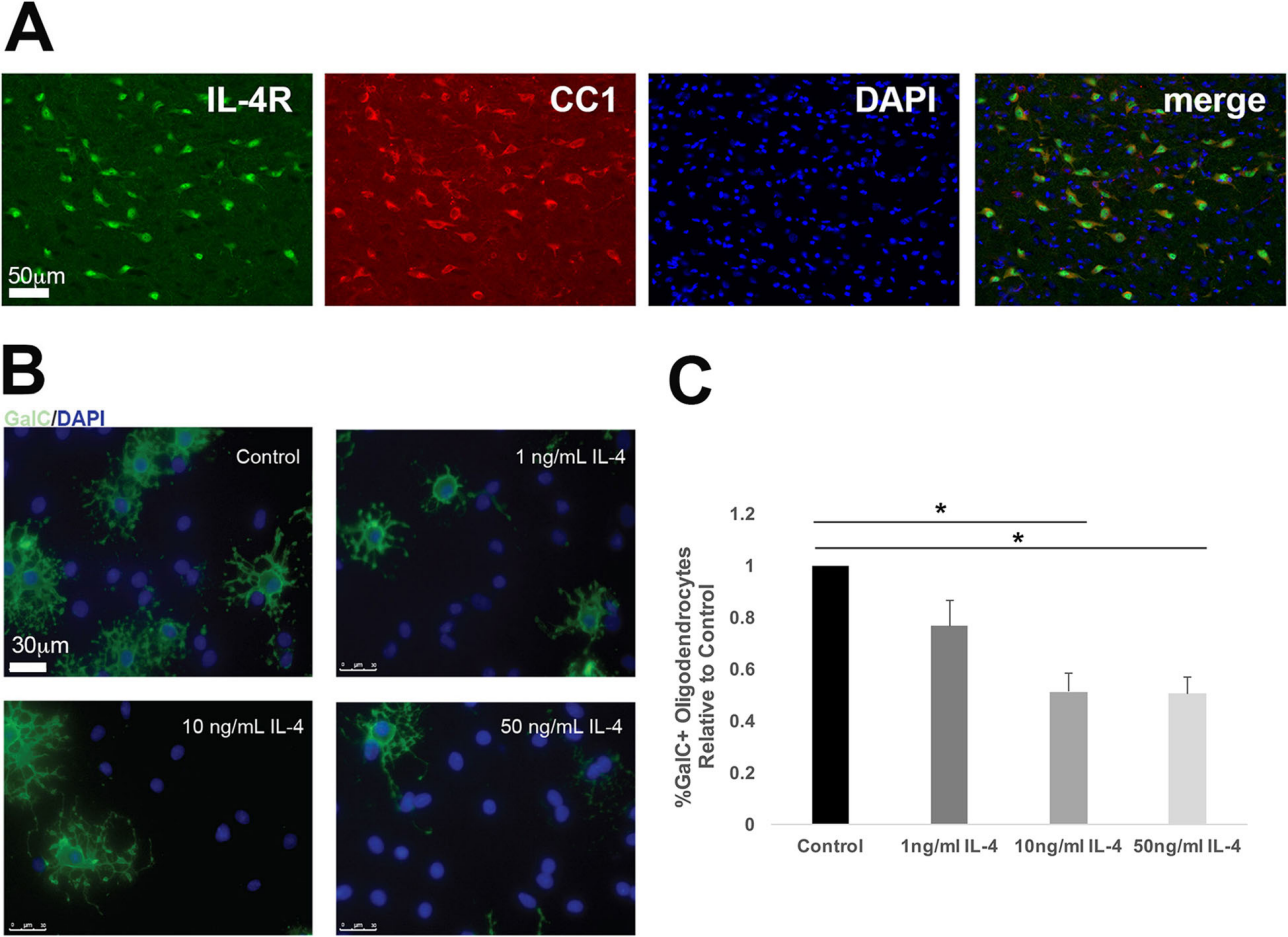
Oligodendrocytes have IL-4 receptors and exhibit decreased differentiation when treated in vitro with IL-4. **a** Sections of normal rat brain at the level of the corpus callosum were double stained with antibody to IL-4 receptor (IL-4R) and CC-1, with DAPI as a nuclear marker. **b** Rat oligodendrocyte progenitors were grown in culture and treated with increasing concentrations of IL-4 at time of differentiation. Staining for GalC, a marker of both immature and mature oligodendrocytes was performed after 72 h and shows a dose-dependent decrease in GalC+ cells. DAPI, blue; GalC, green. Size bar = 30 μm. **c** Quantification of percent differentiation, GalC+ cells relative to DAPI+ nuclei, in rat OPCs treated with IL-4 and control (*n* = 3 biological replicates). *Significant difference versus control (1 ng/ml: *P* = 0.100, 10 ng/ml: *P* = 0.013, 50 ng/ml: *P* = 0.023), two-tailed *T* test

Little to no data exists about the effects of IL-4 directly on oligodendrocytes in vitro. However, there are multiple studies showing that oligodendrocytes have IL-4 receptors [15, 26]. We labeled cells in vitro and in the corpus callosum of PD14 rats with antibody to the IL-4 receptor and an oligodendrocyte marker and detected labeling in oligodendrocytes in both (in vivo labeling, Fig. 6a, in vitro: not shown). We also examined the effect of IL-4 directly on primary oligodendrocytes during differentiation over a 72-h period (Fig. 6b, c). IL-4 inhibited differentiation in all concentrations tested. GalC+ cells were quantified with respect to total cells. The most robust decrease occurred at 50 ng/ml in which treated cells had 66% fewer GalC+ cells than controls. Nuclear morphology by DAPI labeling showed that cell death was not induced at these concentrations.

## Discussion

Growth-restricted newborns are at high risk for neonatal mortality as well as motor deficits, behavioral deficits, and cerebral palsy due, at least in part, to lack of proper myelination [2–4]. In this study, we used a well-tested model of uteroplacental insufficiency in which we have previously demonstrated a significant developmental delay in oligodendrocyte maturation and myelination as well as behavioral defects in the adult [5]. Previous studies using the same model showed that the reduction in myelination was due to inhibition of maturation as numbers of oligodendrocyte precursors were equal between control and IUGR [5]. Here, we showed that inflammation is a significant factor in the etiology of the myelin deficits in IUGR pups. We identified a number of cytokines and chemokines that are modulated as a result of IUGR and showed that resident microglia/macrophages are increased in number and become activated. One cytokine in particular, IL-4, was significantly increased. Although IL-4 is usually thought to be protective, our results using an IL-4 neutralizing antibody demonstrated that excess IL-4 in the newborn damaged oligodendrocytes and impaired myelin development. Blocking IL-4 protein immediately after birth rescued the oligodendrocyte and myelin phenotype. Treatment of primary cultures of oligodendrocytes with IL-4 also showed inhibition of differentiation, suggesting that IL-4 could directly mediate these effects.

Inflammation is now recognized as playing a fundamental role in the pathogenesis of many myelin disorders such as multiple sclerosis. However, it is generally accepted that multiple sclerosis is primarily a Th1 disease, although a recent clinical study has demonstrated that patients with progressive multiple sclerosis as well as relapsing-remitting patients have increased IL-4 levels [27]. Although Th2 responses and IL-4 are thought to be protective in the adult brain, priming of macrophages with IL-4 followed by a pro-inflammatory stimulation can result in an enhanced inflammatory response [17]. Multiple studies have also demonstrated that growth-restricted newborns have increased inflammatory proteins both in their serum and cord blood when compared with those appropriate for gestational age [7, 8]. A recent transcriptome analysis performed on oligodendrocytes and microglia/macrophages from a rat malnutrition model showed dysregulation of a number of neuroinflammatory genes [28]. Using our uteroplacental insufficiency model [5, 11, 20], which induces hypoxia and leads to fetal growth restriction, we found a localized Th2 response in the rat brain peaking postnatally at a time which coincides with developmental myelination. Previous studies [5] have demonstrated oligodendrocyte and myelin injury in IUGR animals, as well as motor deficits similar to those observed in children who were IUGR at birth [3, 29, 30]. While the etiology of white matter injury in IUGR has been poorly understood, our novel results demonstrate a mechanistic link between Th2 inflammation and white matter injury.

Inflammation in the brain can be mediated through microglia, the resident macrophages of the CNS. During prenatal brain development, microglia are located in areas susceptible to white matter injury such as the corpus callosum and can be the source of inflammatory cytokines, which have been shown to be damaging to oligodendrocytes in cell culture models [31]. Other inflammatory cells such as macrophages and T cells may also invade the newborn brain to cause damage. We have shown that markers of Th2 inflammation including IL-4 were increased in the IUGR rat brain. This inflammation increased microglia/macrophages in the corpus callosum and areas of white matter injury, leading to a 2.5-fold increase in microglia/macrophages. Although the increase in microglia/macrophages has been observed before in other IUGR models, none of these studies elucidated immune or inflammatory mechanisms [28, 32, 33]. In our study, the microglia/macrophages were larger than in control animals and were in an activated state, which may further contribute to white matter injury. The total brain weight in IUGR animals was equivalent to that in control, indicating that volume loss was not contributing to the apparent increase in microglia/macrophages (data not shown).

Based on our Luminex data and previous studies [11] and to determine causality of IL-4 to white matter injury, we utilized a treatment course of neutralizing IL-4 antibody injection immediately after birth. Previous work by Jaeckle Santos et al. demonstrated that this antibody is specific to IL-4, and treatment with a non-specific IgG antibody did not ameliorate the IUGR phenotype present in β cells [11]. Anti-IL-4 treatment decreased IL-4 and resulted in increased numbers of mature oligodendrocytes and increased myelin proteins which were restored to or nearly to the level of controls. The most robust result was seen in levels of MBP. More modest but still significant results were seen with PLP and CNP. Since CNP expression occurs very early in the course of oligodendrocyte differentiation [34], it is possible that its expression was already partly established before IL-4 exerts its effect. Although these animals will eventually develop diabetes which is corrected with IL-4 injection, the hypoglycemia in the IUGR animals does not appear until adulthood [11], and thus, the effects on myelination in this model were direct and not due to rescue of the metabolic syndrome. The rescue of myelination may potentially lead to improvements in cognition and motor deficits later in life. In contrast to our findings in the newborn, previous studies in adult stroke models have shown that IL-4 is helpful for functional recovery [35–37]. Further, in tissue culture experiments and in the experimental autoimmune encephalomyelitis (EAE) model of multiple sclerosis, treatment of microglia/macrophages with IL-4 promoted the generation of oligodendrocytes [38, 39]. Thus, our data strongly suggest that the neuroimmune response in the fetus and newborn is very different from that in the adult and that there is an exaggeration of the Th2 response in the perinatal period, which is damaging to the IUGR brain. The finding that the newborn neuroimmune response is more robust than in the adult has been shown in a number of models, especially in hypoxic ischemic injury, in which there was increased cytokine production in the newborn and a heightened response to those cytokines compared with older animals [40–44]. Likewise, the finding that the role of a cytokine or growth factor in neural cells can switch from damaging to protective with age has been shown by Kim et al. using an inhibitor of TGF-β1 signaling [43].

Prior to this study, little data existed on the direct action of IL-4 on oligodendrocytes, although direct action on microglia/macrophages and astrocytes has been reported as noted above. Human oligodendrocytes have IL-4 receptors which are upregulated during MS and oligodendrocytes also express STAT6, a downstream target of IL-4 signaling, and STAT6 expression is increased in MS [15]. IL-4 receptors have been detected on rat OPCs and oligodendrocytes by q-PCR although expression of the receptors was more robust on astrocytes in the same species [15, 19, 45, 46]. In our studies, direct treatment of primary rat oligodendrocyte cultures with IL-4 showed decreased differentiation compared with control, in support of our in vivo results. This is in contrast to a report by Paintlia et al. [47] who demonstrated in vitro that the negative effects of LPS on oligodendrocyte maturation could be blocked by IL-4. In the course of these experiments, their data also suggested that oligodendrocyte differentiation might be potentiated in vitro by IL-4 [47]. This discrepancy maybe explained by culture conditions or levels of cytokines used. Our studies do not rule out that IL-4 may affect oligodendrocytes indirectly through other cell types in vivo. Further studies will be needed to determine the intracellular pathway by which IL-4 regulates oligodendrocyte differentiation. Several brain-derived growth factors, such as members of the bone morphogenetic protein (BMP), Wnt or notch families, inhibit oligodendrocyte differentiation during development and are upregulated during demyelinating or dysmyelinating disease [5, 48, 49], and IL-4 may cooperate with one of these or employ a novel pathway.

It is not clear yet which cell type(s) is currently making IL-4 or if oligodendrocytes specifically from IUGR rats are more susceptible to IL-4 damage. It is possible that innate brain cells such as microglia or astrocytes are producing IL-4, but it is also possible invading immune cells such as macrophages, eosinophils, or basophils are also producing IL-4. This is supported by the elevations of eotaxin (an eosinophil chemoattractant) in our Luminex data. Identifying the cells of origin is of interest and will be the subject of study in future studies.

## Conclusions

In summary, we have shown IUGR induces a localized exaggerated Th2 inflammatory response which is causal to oligodendrocyte and white matter injury. This is the first demonstration that IL-4 works to inhibit oligodendrocyte differentiation and function in the newborn animal. In addition, it is clear that a Th2 response previously thought to be helpful in adult stroke models is drastically different in the fetus and newborn, leading to increased injury. These results will allow us to develop new therapeutic modalities for treatment of white matter injury affecting many newborns.

## Data Availability

The datasets used and/or analyzed during the current study are available from the corresponding author on reasonable request.

## Abbreviations

CNP: 3′,5′-Cyclic nucleotide phosphodiesterase
DAPI: 4′,6-Diamidino-2-phenylindole
GalC: Galactocerebroside
GAPDH: Glyceraldehyde 3-phosphate dehydrogenase
GFAP: Glial fibrillary acidic protein
IUGR: Intrauterine growth restriction
MBP: Myelin basic protein
OPC: Oligodendrocyte progenitor cell
PBST: Pospho-buffered saline with Tween
PD: Postnatal day
PLP: Proteolipid protein
TBS: Tris buffered saline
TBST: Tris buffered saline with Tween
